# District Nurses and Their Work

**Published:** 1916-10-28

**Authors:** 


### THE MATRONS' AND SISTERS' DEPARTMENT.

### DISTRICT NURSES AND THEIR WORK.

THE term "district nurse," as commonly used, has so varied a meaning that it may be useful to indicate the different grades of nurses included in it. First, there is the fully-trained Queen's nurse, who, in addition, usually possesses the midwifery certificate, and has always had six months' training under experienced superintendents in actual nursing in working-class homes. Another class of "district nurse" includes those who may, or may not, have had three years' hospital training, but who are not Queen's nurses—*i.e.* they have not had the special six months' training to entitle them to be put on the Roll of Queen's Nurses. Thirdly, there is the village nurse. This group of "district nurse" has not necessarily had any hospital training at all, but merely twelve months' district and midwifery training under trained nurses. She thus never sees nursing under good conditions. She is supposed to work in the country districts, and chiefly as a midwife. The fourth class of "district nurse" is the cottage nurse, with three to six months' district training. These nurses live in the cottages and should be called cottage helps and not nurses.

Originally the work of the district nurse was to nurse the sick poor in their own homes. It now includes preventive work (and in their training insistence is laid on the opportunities district nurses have not only of preventing the spread of disease but of preventing disease at all), midwifery, assisting the doctor in school medical inspection and visiting the children and their parents in their own homes, as well as nursing and supervising tubercular patients under the National Insurance Act. More recently the Notification of Births Act and the institution of mothers' clubs and babies' welcomes involve the necessity for health visiting, and now, as the result of the war, there will be scattered over the United Kingdom many thousands of men in their own homes, some needing skilled nursing attention for the rest of their lives, others needing it for a considerable length of time. Who is to do all this additional work in the future? The district nurse seems the most suitable person, but under present conditions the term "district nurse" covers too great a variety of training and of lack of training to ensure the skilled nursing required.

The question is, How is this skilled nursing to be obtained for these men in their own homes? In most counties there are far more village nurses (or midwives, as they should be called) than there are fully-trained or "Queen's" district nurses. The village nurse is cheaper than the "Queen's": she has had a much less expensive training, and her social circumstances enable her to live more cheaply. There is thus a difficulty in getting enough fully-trained nurses to become Queen's nurses. Why is this? The work now cannot be considered monotonous, neither can a woman who has such a variety of work to do become narrow; the real reasons are probably the small pay, the difficulties of getting about (usually in scattered districts on a cycle in all weathers), and the fact that so few matrons of training schools know anything of the work of district nurses that they do not encourage the best type of nurse to do this work. In fact, many matrons discourage any but the uneducated and the least capable taking up this branch of nursing, which surely requires the highest standard, both in general and nursing education, and the greatest capacity.

The salary question is an important one. A Queen's nurse usually starts at £90, inclusive; this means that, after having had three years' hospital training, six months' district training, four months' midwifery, and probably qualifying also for a health visitor's, or sanitary inspector's, or school nurse's certificate, none of which can be obtained without the expenditure of time and money, the nurse is left with £30 as salary, the remaining £60 having to go in board, lodging, laundry, and uniform. To obtain a sufficient supply of well-trained, well-educated nurses for this branch of nursing—and such women must be obtained if the work mentioned is to be properly done—the salaries ought to begin at £120, rising to £150, with well-paid superintendents' and inspectors' posts for those who are suitable for and care about organising and supervision work. How is this money to be raised so that every village and hamlet can have the services of trained district nurses? One of the ways suggested is that larger grants should be made by the Education Authorities, National Health Insurance Committees, Tuberculosis Committees, and various other public bodies. The getting of subscriptions from the charitable and also from the patients should be of course continued, so that the local district nursing committee would be as hitherto responsible for raising by voluntary means a certain amount of the sum required. Another suggestion is that district nurses should be paid, as other public servants are paid, either out of the county rates or direct by the State.

